# Stent-Screw Assisted Internal Fixation of Osteoporotic Vertebrae: A Comparative Finite Element Analysis on SAIF Technique

**DOI:** 10.3389/fbioe.2019.00291

**Published:** 2019-10-25

**Authors:** Luigi La Barbera, Alessandro Cianfoni, Andrea Ferrari, Daniela Distefano, Giuseppe Bonaldi, Tomaso Villa

**Affiliations:** ^1^Laboratory of Biological Structure Mechanics, Department of Chemistry, Materials and Chemical Engineering “G. Natta,” Politecnico di Milano, Milan, Italy; ^2^Department of Mechanical Engineering, Polytechnique Montréal, Montreal, QC, Canada; ^3^Sainte-Justine University Hospital Centre, Montreal, QC, Canada; ^4^Department of Neuroradiology, Neurocenter of Southern Switzerland, Lugano, Switzerland; ^5^Department of Interventional and Diagnostic Neuroradiology, Inselspital, University Hospital of Bern, Bern, Switzerland; ^6^Neurochirurgia - Casa di Cura Igea, Milan, Italy

**Keywords:** osteoporosis, vertebral compression fractures (VCF), finite element model (FEM), screw-stent assisted internal fixation (SAIF), spine biomechanics, vertebral augmentation

## Abstract

Vertebral compression fractures are one of the most relevant clinical consequences caused by osteoporosis: one of the most common treatment for such fractures is vertebral augmentation through minimally invasive approaches (vertebroplasty or balloon-kyphoplasty). Unfortunately, these techniques still present drawbacks, such as re-fractures of the treated vertebral body with subsidence of the non-augmented portions or re-fracture of the non-augmented middle column at the junction with the augmented anterior column. A novel minimally-invasive augmentation technique, called Stent-Screw Assisted Internal Fixation, has been recently proposed for the treatment of severe osteoporotic and neoplastic fractures: this technique uses two vertebral body stents and percutaneous cannulated and fenestrated pedicular screws, through which cement is injected inside the expanded stents to achieve optimal stents' and vertebral body's filling. The role of the pedicle screws is to anchor the stents-cement complex to the posterior column, acting as a bridge across the middle column and preserving its integrity from possible collapse. In order to evaluate the potential of the new technique in restoring the load bearing capacity of the anterior and middle spinal columns and in reducing bone strains, a Finite Element model of an osteoporotic lumbar spine has been developed. Both standard vertebroplasty and Stent-Screw Assisted Internal Fixation have been simulated: simulations have been run taking into account everyday activities (standing and flexion) and comparison between the two techniques, in terms of strain distribution on vertebral endplates and posterior and anterior wall, was performed. Results show that Stent-Screw Assisted Internal Fixation significantly decrease the strain distribution on the superior EP and the cortical wall compared to vertebroplasty, possibly reducing the re-fracture risk of the middle-column at the treated level.

## Introduction

Osteoporosis, defined as “a systemic skeletal disease characterized by low bone mass and micro-architectural deterioration of bone tissue with a resultant increase in fragility and risk of fracture,” is a major clinical issue worldwide (Lippuner, 2003). Vertebral compression fractures (VCFs) is one of the most relevant clinical consequences, potentially causing acute and chronic pain, and reduced quality of life (Du et al., 2014), with an impact on mortality (Edidin et al., 2015). VCFs can occur spontaneously or due to trauma, generally a compressive load injury mechanism involving the vertebral body (VB) (Ensrud and Schousboe, 2011). The anterior and middle vertebral columns together support about 80% of the overall spinal load in standing, and those are most commonly involved (White and Panjabi, 1990). The spectrum of severity may range from mild and stable compression fractures, affecting the disc-endplate (EP) region and leading only to minor deformity, to unstable fractures with a high-degree of osseous fragmentation, collapse deformity, middle column involvement, pediculo-somatic junction fracture, and kyphotic deformity (Denis, 1983; Genant et al., 1993; McCormack et al., 1994).

Vertebral augmentation (VA), performed with vertebroplasty or balloon-kyphoplasty, implies percutaneous image-guided injection of bone cement in the anterior two thirds of the VB (i.e., the anterior column), and it is widely used to treat fragility fractures, to arrest fracture progression, to palliate pain and to restore the load-bearing capability of the VB (Wardlaw et al., 2009; Klazen et al., 2010; Firanescu et al., 2011; Clark et al., 2016; Filippiadis et al., 2017). The injection of cement in the VB aims at a homogeneous trabecular filling, but it is stopped for safety reasons, when the cement approaches the posterior third of the VB, to avoid leakage in the central canal.

Re-fracture of the treated VB is a well-known and reported event following VA, although its timing and frequency are variable among published reports (Lin et al., 2008; Li et al., 2018). The re-fracture usually implies subsidence of the non-augmented portions of the VB around the cement cast (Nagaraja et al., 2015). This event may lead to minimal adjustment of the adjacent bony structures or it may lead to extensive collapse of the non-augmented portions of the vertebra.

A less frequent event is the re-fracture of the non-augmented middle column at the junction with the augmented anterior column (Gan et al., 2014). These fractures are often characterized by collapse and retropulsion of the posterior wall, eventually associated with catastrophic splitting and separation between the augmented anterior portion of the VB and the middle column, accompanied by focal kyphotic deformity. Although largely under-reported in the literature, these dramatic events pose a real therapeutic challenge (Abudou et al., 2013; Gonschorek et al., 2017).

The importance of the mechanical stability of the middle column might be largely underestimated, since the load-bearing capacity of the vertebra is usually referred just addressing the anterior column. Furthermore, the middle column, with the posterior third of the VB, the posterior wall, and the pediculo-somatic junctions might represent a weak region even after satisfactory VA. In fact, it is expected that local strain gradients across the stiffer augmented and the weaker non-augmented regions, may lead to intensification effects, exposing to the risk of a secondary middle column re-collapse. This event may be particularly dramatic in severely osteoporotic patients or following a first severe “burst fracture” involving the anterior and middle columns.

**Figure 1:**
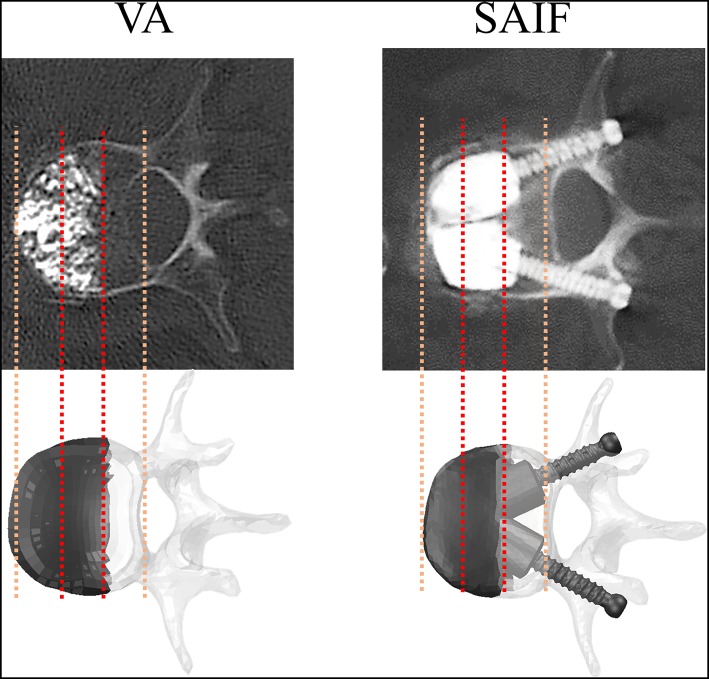
Post-operative CT images of vertebrae treated with VA and SAIF techniques compared with the simulated ones: in both cases, cement filling involves 2/3 of the vertebral body and it is anteriorly located. CT images are courtesy of A.C.

A novel minimally-invasive augmentation technique, called Stent-Screw Assisted Internal Fixation (SAIF, Figure 1) has been recently proposed by Cianfoni et al. for the treatment of severe osteoporotic and neoplastic fractures (Cianfoni et al., 2019a,b). The SAIF technique includes insertion and balloon-expansion of two vertebral body stents (VBS), followed by the insertion of percutaneous cannulated and fenestrated pedicular screws. After the stents are expanded and the screws are in position, the cement is injected through the screws to achieve optimal stents' and VB's filling (endplate-to-endplate). The role of the stents is to help maintain the height restoration achieved by balloon inflation, avoiding deflation effect, and to act as a scaffold that allows homogeneous anterior column augmentation and prevents cement leakage (Rotter et al., 2010; Diel et al., 2013; Cianfoni et al., 2019b).

The potential role of the pedicle screws is to anchor the VBS-cement complex to the posterior elements, avoiding its displacement, and to act as a bridge across the middle column, preserving its integrity from possible collapse and splitting (Cianfoni et al., 2019a). As such, SAIF technique might reduce the risk of middle column collapse after a VA treatment in severe osteoporotic vertebral fractures.

Different studies investigated the relative importance of biomechanical factors playing a role in VA techniques. Rohlmann et al. (2010) performed a probabilistic numerical study reporting that in an augmented vertebra the cement volume and its elastic modulus have a dominant role compared to shape and symmetry of the cement plugs. Chevalier et al. (2008) demonstrated that cement bridging both endplates (EPs) restores the load–bearing capacity of the treated vertebra (i.e., its vertebral stiffness and strength). Ottardi et al. (2016a) demonstrated that a full height restoration is a key factor in reducing the stress on the surrounding structures.

A recent biomechanical study demonstrated the effectiveness of SAIF technique in restoring the load-bearing capacity of an extensively lytic vertebra, while reducing the strains (i.e., fracture risk) on surrounding bony structures (La Barbera et al., 2019). However, there are no studies investigating the SAIF technique in an osteoporotic model.

The aim of the current computational comparative study was to investigate whether SAIF technique is biomechanically advantageous compared to standard VA in restoring the load bearing capacity of the anterior and middle spinal columns and in reducing bone strains, in a lumbar spine osteoporotic model.

## Materials and Methods

### Intact OP Model

**Figure 2:**
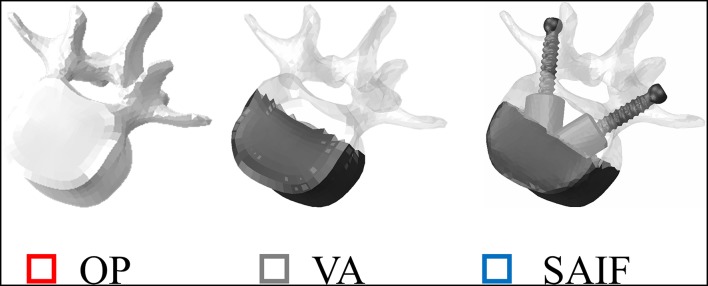
Representation of L3 vertebrae in all simulated conditions. From left to right: osteoporotic vertebra (OP) taken as a reference condition, vertebral augmentation (VA) and the new Stent-Screw Assisted Internal Fixation (SAIF). Bone is highlighted in shaded white, while screws and bone cement are in dark grey.

An intact non-linear FEM describing the L1-S1 spine segment of a healthy 40 years-old human male without any spinal defect was initially considered (Ottardi et al., 2016b). The model (Figure 2), complete of vertebral bodies, intervertebral discs and 7 groups of lumbar ligaments, has already been validated by comparison with experimental measurements considering its kinematics, the compressive stiffness of the vertebrae and the strains reached on the cortical bone of the VB (Ottardi et al., 2016a).

Material properties were assumed from literature, as reported in a previous validation study (Ottardi et al., 2016b). To properly simulate an osteoporotic condition, the mechanical properties of the cancellous and cortical vertebral bone were reduced according to literature data for each VB (Chae et al., 2010). The model thus created was herein named “OP model.”

To prevent any artifact due to the application of the boundary conditions at cranial and caudal levels, the middle vertebra (L3) was selected as the level of interest to reproduce the different surgical techniques.

### VA Model

**Figure 3:**
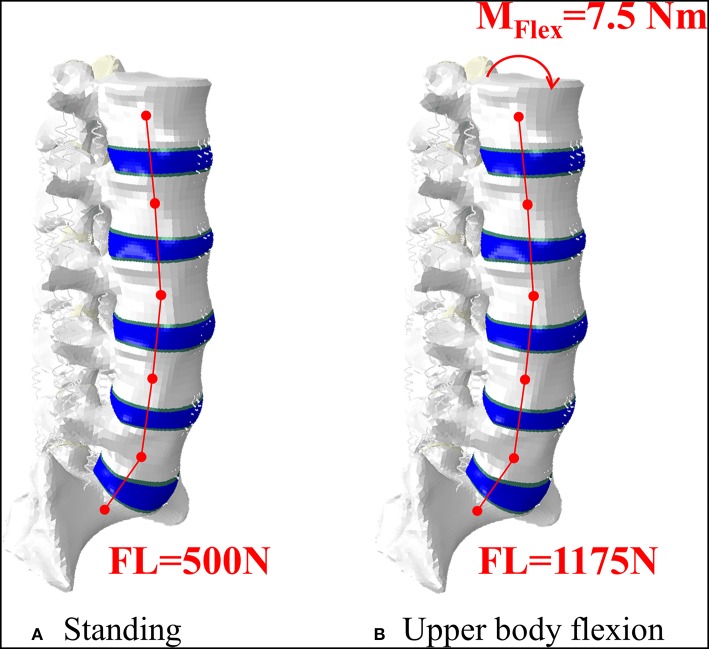
Representation of the intact model in standing, where an axial follower load (FL) was applied **(A)**, and in upper body flexion, where an additional bending moment was applied on the superior EP of L1 **(B)**. The lower part of S1 was constrained in both conditions.

The vertebral augmentation (VA) technique was simulated by increasing the elastic modulus of anteriorly located elements from osteoporotic bone to cement. Such elements cover 2/3 of the whole L3 VB volume, according to post-operative imaging (Figures 1, 3). The cement volume (about 20 ml) resulted from the choice to reproduce optimal endplate bridging (Chevalier et al., 2008).

### SAIF Model

To describe SAIF technique on the OP model, the CAD model of the cannulated pedicle screw (2B1 SRL, Milan, Italy) was properly assembled in the two pedicles of the L3 vertebra using ICEM CFD (Ansys Inc.), following boolean operations, the whole vertebra was finally remeshed using linear tetrahedral elements. Attention was paid in maintaining a good compromise between adequate mesh refinement and reasonable computational cost. For the same reason, the metallic stent was not included in the model, assuming it gives a negligible contribution to the overall compressive stiffness of the treated vertebra, since the injected bone cement usually completely fills and surrounds the stents: however, the contribution of the cement confined into the stents was taken into account by creating two PMMA cylinders around the screws that simulate the stents filled with PMMA cement (Figures 1, 3). To evaluate the full potential of SAIF technique, optimal endplate-to-endplate cement augmentation and maximal height restoration were assumed.

For all the materials linear elastic properties were assumed (Table 1), for the remaining properties (not modified from the original model) the reader is addressed to Ottardi et al. (2016b).

**Table 1:** Mechanical properties of the materials used in the simulations. For other material properties, please refer to Ottardi et al. (2016b).

	**Type of material**	**Elastic modulus (MPa)**	**Poisson ratio (–)**	**References**
Osteoporotic cancellous bone	Transversely isotropic	123.2 123.2 176	0.45 0.32 0.32	Chae et al., 2010; Ottardi et al., 2016a
Osteoporotic cortical bone	Linear isotropic	4,320	0.3	Ottardi et al., 2016a
Bone cement (PMMA)	Linear isotropic	2,500	0.438	Hansen and Jensen, 1992
Titanium (pedicle screw)	Linear isotropic	110,000	0.3	La Barbera et al., 2015, 2017; La Barbera and Villa, 2016

### Loading Conditions

All models underwent two different loading scenarios (Figure 2). Standing was simulated applying a 500N follower load (Rohlmann et al., 2009; La Barbera et al., 2016b, 2017). Flexion of the upper body, often associated to the event of VCF, was reproduced with a 1175N follower load and a 7.5 N/m moment on the L1 vertebra (Rohlmann et al., 2009; La Barbera et al., 2016b, 2017). In both cases the inferior portion of S1 was considered fully constrained.

All the simulations were run on ABAQUS Standard 2017 (Dassault Systèmes Ri, Simulia Corp, Providence, RI, USA).

### Comparative FE Analyses

The load distribution in the L3 vertebra for the untreated osteoporotic (OP) condition, and for both techniques (VA, SAIF) was evaluated in terms of maximum and minimum principal strains on the cortical regions. Principal strains values, possibly related to bone fracture risk (Imai, 2015; Palanca et al., 2018; Wáng et al., 2018), were evaluated at nodal values in specific regions located on the endplates, anterior and posterior walls. The endplates were divided in two regions of interest: the anterior and the middle column, corresponding to the cortical bone laying above the cement and the osteoporotic bone, respectively (specific elements were excluded to avoid strain intensification effects occurring at cement-bone interface). To highlight any statistical difference between the median values collected on each region of interest a paired Wilcoxon test with a 0.05 significance level was performed. Box plot representation, showing 25–75% interquartile ranges, median bar and whiskers indicating the 5–95% range (with a cross indicating the average value), was used to allow qualitative comparison.

To point out any mechanical issue related to the usage of the cannulated pedicle screw in all different scenarios, the maximum von Mises stresses was also considered.

## Results

The median values obtained on the anterior column demonstrate that both SAIF and VA techniques reduced the principal strains in the treated vertebra compared to the OP case (Table 2).

**Table 2:** Median principal strains values obtained in all regions of interest of the treated vertebra (L3) both for standing and upper body flexion on OP, VA, and SAIF models.

**Region of interest**			**Standing**						**Upper body flexion**						**Flexion vs. standing**		
			**OP**	**VA**	**SAIF**	**VA vs. OP**	**SAIF vs. OP**	**SAIF vs. VA**	**OP**	**VA**	**SAIF**	**VA vs. OP**	**SAIF vs. OP**	**SAIF vs. VA**	**OP**	**VA**	**SAIF**
Anterior column	Superior EP	Max princ. strains (%)	0.018	0.010 (^*^)	0.002 (^*^, ^†^)	**−*44***%	**−*89***%	**−*80***%	0.059	0.025 ^*^	0.008 (^*^, ^†^)	**−*58***%	**−*8*6**%	**−*68***%	*228*%	*150*%	*300*%
		Min princ. strains (%)	**–**	**–**	**–**	**–**	**–**	**–**	**–**	**–**	**–**	**–**	**–**	**–**	**–**	**–**	**–**
	Anterior wall	Max princ. strains (%)	0.020	0.004 (^*^)	0.002 (^*^, ^†^)	**−*80***%	**−*90***%	**−*50***%	0.075	0.020 (^*^)	0.011 (^*^, ^†^)	**−*73***%	**−*86***%	**−*45***%	*275*%	*400*%	*450*%
		Min princ. strains (%)	−0.036	−0.013 (^*^)	−0.008 (^*^, ^†^)	**−*64***%	**−*78***%	**−*39***%	−0.155	−0.065 (^*^)	−0.036 (^*^, ^†^)	**−*58***%	**−*77***%	**−*45***%	**–**	**–**	**–**
	Inferior EP	Max princ. strains (%)	0.016	0.009 (^*^)	0.003 (^*^, ^†^)	**−*44***%	**−*81***%	**−*67***%	0.053	0.025 (^*^)	0.010 (^*^, ^†^)	***−53***%	***−81***%	**−*60***%	*231*%	*178*%	*233*%
		Min princ. strains (%)	**–**	**–**	**–**	**–**	**–**	**–**	**–**	**–**	**–**	**–**	**–**	**–**	**–**	**–**	**–**
Medial column	Superior EP	Max princ. strains (%)	0.027	0.023 (^*^)	0.015 (^*^, ^†^)	**−*15***%	**−*44***%	**−*35***%	0.035	0.028 (^*^)	0.020 (^*^, ^†^)	**−*20***%	**−*43***%	**−*29***%	*30*%	*22*%	*33*%
		Min princ. strains (%)	−0.019	−0.022	−0.010 (^*^, ^†^)	*+16*	**−*48***%	**−*55***%	−0.018	−0.021	−0.012 (^*^, ^†^)	+17%	**−*33***%	**−*43***%	**–**	**–**	**–**
	Posterior wall	Max princ. strains (%)	0.025	0.013 (^*^)	0.007 (^*^, ^†^)	**−*48***%	**−*72***%	**−*46***%	0.036	0.011 (^*^)	0.004 (^*^, ^†^)	**−*69***%	**−*89***%	**−*64***%	*44*%	−*15*%	−*43*%
		Min princ. strains (%)	−0.032	−0.022 (^*^)	−0.021 (^*^)	**−*31***%	**−*34***%	−*5*%	−0.046	−0.018 (^*^)	−0.017 (^*^)	**−*61***%	**−63**%	*−6*%	**–**	**–**	**–**
	Inferior EP	Max princ. strains (%)	0.024	0.020	0.022	*−17*%	*−8*%	*−4.5*%	0.033	0.018 (^*^)	0.024 (^*^)	**−*46***%	**−*27***%	+*33*%	*38*%	−*10*%	*9*%
		Min princ. strains (%)	−0.007	−0.005	−0.014	*−29*%	*+100*%	*+180*%	−0.011	−0.004	−0.017	*−64*%	*−55*%	*+325*%	**–**	**–**	**–**

*Percentage differences for VA vs. OP and for SAIF vs. OP and vs. VA are highlighted in bold, whenever differences are significant. To quantify variations in load sharing due to flexion, percentage strain increase compared to standing are also provided*.

*,† *Significant differences (p < 0.05) in median values compared to OP and VA models, respectively*.

Negligible compressive strain values (with absolute value < 0.001) are not reported and are here indicated with “–.”

### Standing

**Figure 4:**
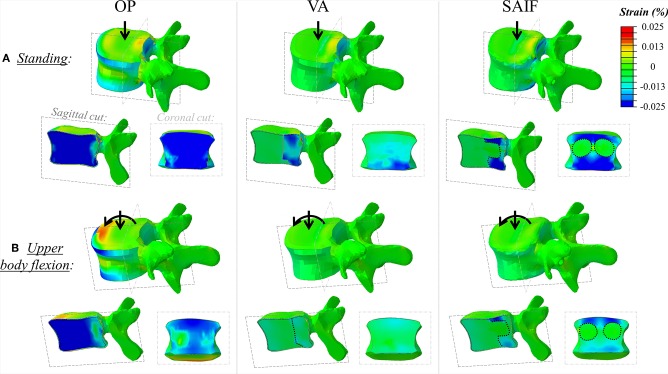
Principal strain maps on L3 vertebra in the untreated OP condition and in VA and SAIF models both in standing **(A)** and in upper body flexion **(B)**. Sagittal cut through the entire vertebra and coronal cut through the middle column are also presented (the dotted lines cuts highlight the contour of the bone cement in VA, while cement it is also distributed around the fenestrated pedicle screw in SAIF to reproduce VBS shape).

The OP model demonstrates rather homogeneous strains across the whole VB, reaching relatively high values. Both EPs and the posterior wall undergo tensile strains due to transversal expansion (Poisson effect) of the trabecular bone which is compressed by the vertical load (Figure 4A).

**Figure 5:**
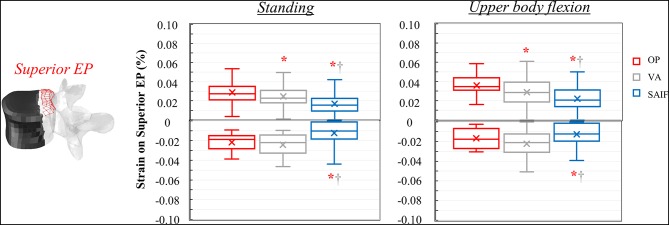
Box plots representing the strains on the superior EP of L3 for all the simulated configurations. The regions of the middle column where the strains were evaluated are highlighted in red on the L3 vertebra. ^*,†^Significant differences (*p* < 0.05) in median values compared to OP and VA.

Following VA, the strains significantly decrease on the middle column, due to the higher load shared by the anterior column filled with stiff cement; in the middle column, the median strains significantly decrease of 15% (*p* = 0.03, Figure 5) on the superior EP and of 48% (*p* < 0.01, Figure 5) on the posterior wall, compared to OP condition. A not significant strain decrease is also observed on the inferior EP (−17% compared to OP model, Figure 7).

**Figure 6:**
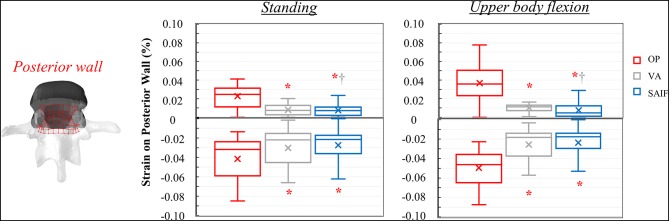
Box plots representing the strains on the posterior wall of L3 for all the simulated configurations. The regions of the middle column where the strains were evaluated are highlighted in red on the L3 vertebra. ^*,†^Significant differences (*p* < 0.05) in median values compared to OP and VA.

Following SAIF, the cannulated transpedicular screw constrains the transversal expansion of the trabecular bone within the middle column, where the remaining trabecular bone results to be loaded in compression similarly to OP case (Figure 4A). Nevertheless, the median strain significantly decreases of 44% (*p* < 0.01, Figure 5) on the superior EP, while of 72% (*p* < 0.01, Figure 6) on the posterior wall compared to OP condition, with an overall significant decrease in strains also compared to VA (superior EP: −35%; posterior wall: −46%, *p* < 0.05).

The maximum Von Mises stresses on the cannulated and fenestrated transpedicular screw in standing was relatively low (18 MPa).

### Flexion

Due to the increased compressive load and the bending moment in flexion, the OP model demonstrates how the load shifts on the anterior column, where both the osteoporotic trabecular bone and the anterior cortical wall reach the highest compressive strains (Figure 4B). In this condition the EPs undergoes tension (Poisson effect). Compared to standing, the anterior column results to be more loaded than the middle one in upper body flexion, with an increase in median strain values of 230% on both EPs and up to 275% on the anterior cortex (Table 2); conversely strain increase are only of 30% up to 44% on the middle column.

**Figure 7:**
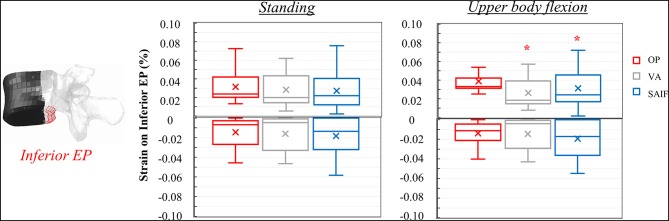
Box plots representing the strains on the inferior EP of L3 for all the simulated configurations. The regions of the middle column where the strains were evaluated are highlighted in red on the L3 vertebra. ^*^Significant differences (*p* < 0.05) in median values compared to OP and VA.

Following VA, the load is shifted even more anteriorly, not only because of the increased load sharing on the augmented anterior spine (stiffer), but also due to the bending moment in flexion. Compared to standing, VA model demonstrates an increase in median strains on the anterior column of 150–178% on the EPs and of 400% on the anterior cortex during flexion (Table 2); the middle column was less affected (+22% on the superior EP, −10% on the inferior EPs, and −15% on the cortex). Compared to OP condition, the median strains on the middle column of VA model were significantly reduced by 20% (*p* = 0.01, Figure 5) on the superior EP, by 46% on the inferior EP (*p* < 0.05, Figure 7), and by 69% (*p* < 0.01, Figure 6) on the posterior wall.

The SAIF model demonstrated the highest strain increase in flexion compared to standing on the anterior column (+230% on the inferior EP, +300% on the superior, +450% on the anterior cortex), while the EPs of the middle column were less affected (+30% on the superior EP, +9% on the inferior) and the posterior wall saw a decrease in strain (−43%). This indicate the capability of SAIF technique in effectively transferring more load than VA on the anterior column, unloading the middle column.

The mechanical role of the cannulated transpedicular screw is to reduce the transversal expansion of the trabecular bone within the middle column compared to OP condition. The resulting strain significantly decreased by 43% (*p* < 0.01, Figure 5) on the superior EP and by 89% (*p* < 0.05, Figure 6) on the posterior wall compared to OP condition, but also compared to VA (−29 and −64%, respectively, *p* < 0.05). Differences between SAIF and VA on the inferior EP were not significant (Figure 7).

The maximum Von Mises stresses on the transpedicular screw slightly increased in flexion, remaining quite low (32 MPa).

## Discussion

Stent-Screw Assisted Internal Fixation (SAIF) technique has been recently introduced by Cianfoni et al. for the treatment of severe osteoporotic and neoplastic fractures (Cianfoni et al., 2019a,b).

SAIF technique couples the clinical advantages typical of VBS (cement augmentation, minimization of leakage, and vertebral height restoration/maintenance) (Cianfoni et al., 2019b) with the percutaneous implantation of cannulated and fenestrated titanium pedicle screws, bridging the augmented VB with the posterior neural arch.

It is interesting to report that other transpedicular implants with or without bone-cement have already been described in the literature for the treatment of osteoporotic VCFs. Kettler et al. reported that BeadEx implant is superior over VA in restoring/maintaining the initial VB height and in providing stability after fracture even following complex dynamic loading *in vitro* (Kettler et al., 2006). Aebi et al. demonstrated that a PEEK V-Strut implant reinforce the VB strength similarly to VA (Aebi et al., 2018). Although purely speculative, the SAIF technique could offer some potential advantages over these techniques. As first, a more adequate reconstruction and scaffolding of the vertebra upon VBS implantation and cement filling, thus maximizing the footprint of the cement within the VB (Cianfoni et al., 2019a). As second, a high biocompatibility typical of titanium alloys of the cannulated screw that can promote bone-integration with the posterior structures.

Although a recent biomechanical study demonstrated the effectiveness of SAIF technique in restoring the load-bearing capacity of an extensively lytic vertebra, while reducing the strains (i.e., fracture risk) on the surrounding structures (La Barbera et al., 2019), no study ever investigated the advantages of SAIF technique in an osteoporotic model. The aim of the present computational study was, therefore, to investigate the advantages of SAIF technique in a lumbar spine osteoporotic model by comparison with standard VA and no treatment (OP). To demonstrate the full potential of the proposed technique, optimal endplate-to-endplate filling was assumed (Chevalier et al., 2008).

Considering standing, our results indicate that SAIF technique is significantly more effective than both no treatment (OP) and simple VA in reducing the median strain distribution across the middle column (Figures 5–7), especially on the superior EP (−44% vs. OP, −35% vs. VA, *p* < 0.05) and on the posterior wall (−72% vs. OP, −46% vs. VA, *p* < 0.05). During upper body flexion, SAIF technique also promotes a higher load transfer on the anterior column compared to simple VA and to the untreated OP condition, while the middle column is less loaded (Table 2). This results in a significant reduction of the median strain across the middle column, especially on the superior EP (−43% vs. OP, −29% vs. VA, *p* < 0.05) and on the posterior wall (−89% vs. OP, −64% vs. VA, *p* < 0.05).

**Figure 8:**
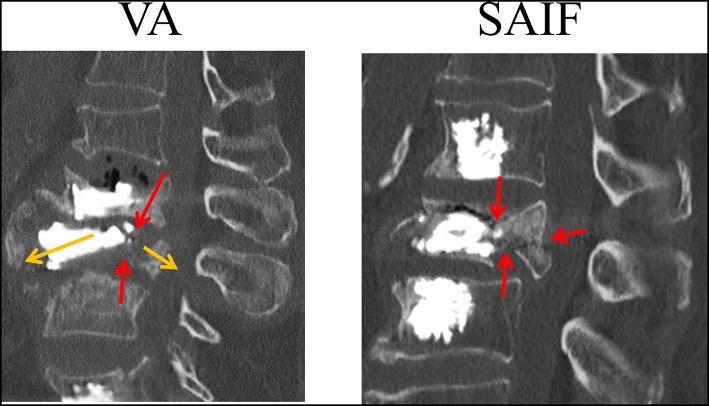
CT image (sagittal slices) taken on a lumbar vertebrae that re-fractured following VA (courtesy of A.C.). The L3 vertebra, previously treated with VA re-fractured with splitting of the anterior and middle column (yellow arrows); it can be noticed that a continuous fracture spreads from the superior to the inferior endplates (red arrows) with posterior wall retropulsion.

The qualitative strain distribution (Figure 4) supports the idea that the presence of convergent pedicle screws constrains the transversal expansion of the trabecular bone in the middle column, thus, reducing the fracture risk in this region compared to simple VA, where the weak not-augmented middle column is substantially “bare” (Cianfoni et al., 2019b). This concept is partially confirmed by post-operative CT images resulting from clinical practice (Figure 8), demonstrating that re-fracture often occurs in the middle column at the treated level following VA due to collapse and splitting. Although from the analysis of these images it is arguable that the weak regions not reinforced by cement are correlated to re-fractures involving the endplates and the posterior wall, it is still not possible to identify where the fracture initially started. Similarly, it is impossible to establish a clear correlation between our findings and the failure mechanisms reported in the published clinical literature (Lin et al., 2008; Li et al., 2018). The simulations performed within our study allowed to investigate one of the leading mechanical factors (i.e., strain distribution) involved in event and to highlight differences between simple vertebral augmentation (VA) and SAIF technique.

Considering the anterior column, SAIF technique is significantly superior to simple VA in decreasing the overall strain distribution, thus, reducing the risk for vertebral collapse (Figure 4, Table 2). This is particularly relevant during upper body flexion (worst-case loading condition) to reduce the fracture risk of the anterior cortex (about −80% vs. OP, about −45% vs. VA, *p* < 0.05) and on both EPs (about −84% vs. OP, about −64% vs. VA, *p* < 0.05). Recalling the assumption of optimal EP-to-EP filling (Chevalier et al., 2008), these results represent a superior limit. Although SAIF allows a more satisfactory reconstruction of the VB compared to VA, suboptimal cement filling of the anterior column may reduce the potential for strain reduction and fracture risk prevention: in this light the simulation of partial filling of the anterior portion of the vertebra and posterior cement filling may represent a surely interesting and valuable development of the present study.

Our study confirmed the mechanical reliability of the cannulated pedicle screw design also for applications in osteoporotic vertebrae. In line with the previous study on SAIF technique in an extensively lytic model (La Barbera et al., 2019), the maximum stress obtained on the cannulated pedicle screws is always much lower than the typical yield strength for titanium alloy (about 750 MPa). This was expected since the screw, as an internal fixation system, does not undergo any relevant loadings typical of standard pedicle screw connected to stiff rigid posterior instrumentation. (La Barbera and Villa, 2016, 2017; La Barbera et al., 2016a,b, 2017).

The present comparative study is surely affected by several limitations. The proposed approach does not describe failure phenomena related to vertebral body collapse. Moreover, the choice of adopting a principal strain criterion (Imai, 2015; Palanca et al., 2018) and of quantitatively analyzing only the cortical structures should be read as a characteristic of the most severe osteoporotic fractures reported by the clinical literature (Genant et al., 1993; Wáng et al., 2018). For the same purpose, namely the choice of simulating only the most severe conditions, the behavior of the system under extension, lateral bending and axial torsion, although surely interesting, was not investigated, because they are considered a less critical loading case with respect to flexion.

The principal strain values, never exceeding the typical failure strains for bone, confirm that the assumption of linear elastic strain is reasonable. In addition, it was assumed that the untreated OP vertebra was not fractured, nor collapsed (with a reduction in anterior height), therefore the results here reported could be considered, ideally, as a preventive cement augmentation, or as the result of a VA following an optimal vertebral height restoration. The choice of not better modeling damage or applying a more complex fatigue prediction is supported by the relatively low values of the calculated principal strains which are well below the static strength of the bone typically reported by the previous literature (Wolfram and Schwiedrzik, 2016). Moreover, the implementation of models correctly describing the peculiarity of a fractured scenario may increase the efforts needed for model validation with *ad hoc* experimental data, while increasing the complexity of the models However, the current approach has the advantage to easily control specific parameters of interest (e.g., screw and cement usage), that may demonstrate a huge variability in clinical practice, adding a confounding effect on the results.

Despite vertebral augmentation techniques have been often related to an increased fracture risk on the vertebral adjacent levels (Ottardi et al., 2016a), such aspect was not analyzed in the current paper. Moreover, despite in clinical practice the adjacent levels might undergo prophylactic vertebral augmentation (Cianfoni et al., 2019a,b), this aspect was not considered in our study and it could be part of future analyses, also evaluating the application of the SAIF technique at other spine levels. Although the results here reported are promising, long-term clinical studies are required to fully demonstrate the safety end the clinical effectiveness of the new SAIF technique over other techniques.

## Conclusions

SAIF technique is biomechanically advantageous over VA in significantly decreasing the strain distribution on the superior EP and the cortical wall, therefore reducing the re-fracture risk of the middle-column at the treated level. The present study provides a strong biomechanical rationale to support the usage of the SAIF technique for the treatment of osteoporotic vertebrae.

## Data Availability Statement

All datasets generated for this study are included in the manuscript.

## Author Contributions

LL designed the study and the FE model. AC, DD, and GB contributed to the study concept and gave clinical input. AF performed the simulations and elaborated the results. TV contributed in designing the study and collected clinical requests. All authors contributed in writing the paper and gave important intellectual contributions to the manuscript, as well as read and approved the final manuscript.

### Conflict of Interest

The authors declare that the research was conducted in the absence of any commercial or financial relationships that could be construed as a potential conflict of interest.

## References

[B1] Abudou M. Chen X. Kong X. Wu T. (2013). Surgical versus non-surgical treatment for thoracolumbar burst fractures without neurological deficit. Cochrane. Database. Syst. Rev. CD005079 10.1002/14651858.CD005079.pub3 23740669 PMC12091996

[B2] Aebi M. Maas C. Di Pauli von Treuheim T. Friedrich H. Wilke H. J. (2018). Comparative biomechanical study of a new transpedicular vertebral device and vertebroplasty for the treatment or prevention of vertebral compression fractures. Clin. Biomech. 56, 40–45. 10.1016/j.clinbiomech.2018.05.001 29803111

[B3] Chae S. W. Kang H. D. Lee M. K. Lee T. S. Park J. Y. (2010). The effect of vertebral material description during vertebroplasty. Proc. Inst. Mech. Eng. H 224, 87–95. 10.1243/09544119JEIM654 20225460

[B4] Chevalier Y. Pahr D. Charlebois M. Heini P. Schneider E. Zysset P. (2008). Cement distribution, volume, and compliance in vertebroplasty: some answers from an anatomy-based nonlinear finite element study. Spine 33, 1722–1730. 10.1097/BRS.0b013e31817c750b 18628704

[B5] Cianfoni A. Distefano D. Isalberti M. Reinert M. Scarone P. Kuhlen D.. (2019a). Stent-screw-assisted internal fixation: the SAIF technique to augment severe osteoporotic and neoplastic vertebral body fractures. J. Neurointerv. Surg. 11, 603–609. 10.1136/neurintsurg-2018-014481 30552168

[B6] Cianfoni A. Distefano D. Pravatà E. Espeli V Pesce G. Mordasini P.. (2019b). Vertebral body stent augmentation to reconstruct the anterior column in neoplastic extreme osteolysis. J. Neurointerv. Surg. 11, 313–318. 10.1136/neurintsurg-2018-014231 30297540

[B7] Clark W. Bird P. Gonski P. Diamond T. H. Smerdely P. McNeil H. P.. (2016). Safety and efficacy of vertebroplasty for acute painful osteoporotic fractures (VAPOUR): a multicentre, randomised, double-blind, placebo-controlled trial. Lancet 388, 1408–1416. 10.1016/S0140-6736(16)31341-1 27544377

[B8] Denis F. (1983). The three column spine and its significance in the classification of acute thoracolumbar spinal injuries. Spine 8, 817–831. 10.1097/00007632-198311000-00003 6670016

[B9] Diel P. Röder C. Perler G. Vordemvenne T. Scholz M. Kandziora F.. (2013). Radiographic and safety details of vertebral body stenting : results from a multicenter chart review. BMC Musculoskelet. Disord. 14:233. 10.1186/1471-2474-14-233 23927056 PMC3751159

[B10] Du J. Li X. Lin X. (2014). Kyphoplasty versus vertebroplasty in the treatment of painful osteoporotic vertebral compression fractures: two-year follow-up in a prospective controlled study. Acta. Orthop. Belg. 80, 477–486. 26280719

[B11] Edidin A. A. Ong K. L. Lau E. Kurtz S. M. (2015). Morbidity and mortality after vertebral fractures: comparison of vertebral augmentation and nonoperative management in the medicare population. Spine 40, 1228–1241. 10.1097/BRS.0000000000000992 26020845

[B12] Ensrud K. E. Schousboe J. T. (2011). Clinical practice. Vertebral fractures. N. Engl. J. Med. 364, 1634–1642. 10.1056/NEJMcp1009697 21524214

[B13] Filippiadis D. K. Marcia S. Masala S. Deschamps F. Kelekis A. (2017). Percutaneous vertebroplasty and kyphoplasty: current status, new developments and old controversies. Cardiovasc. Intervent. Radiol. 40, 1815–1823. 10.1007/s00270-017-1779-x 28856402

[B14] Firanescu C. Lohle P. N. de Vries J. Klazen C. A. Juttmann J. R. Clark W.. (2011). A randomised sham controlled trial of vertebroplasty for painful acute osteoporotic vertebral fractures (VERTOS IV). Trials 12:93. 10.1186/1745-6215-12-93 21466679 PMC3083362

[B15] Gan M. Zou J. Zhu X. Wang G. Yang H. (2014). Balloon kyphoplasty for OP spinal fractures with middle column compromise. Injury 45, 1539–1544. 10.1016/j.injury.2014.06.018 25022230

[B16] Genant H. K. Wu C. Y. van Kuijk C. Nevitt M. C. (1993). Vertebral fracture assessment using a semiquantitative technique. J. Bone. Miner. Res. 8, 1137–1148. 10.1002/jbmr.5650080915 8237484

[B17] Gonschorek O. Hauck S. Weiß T. Bühren V. (2017). Percutaneous vertebral augmentation in fragility fractures: indications and limitations. Eur. J. Trauma. Emerg. Surg. 43, 9–17. 10.1007/s00068-016-0753-7 28101655

[B18] Hansen D. Jensen J. S. (1992). Mixing does not improve mechanical properties of all bone cements. Manual and centrifugation-vacuum mixing compared for 10 cement brands. Acta. Orthop. Scand. 63, 13–18. 10.3109/17453679209154841 1738962

[B19] Imai K. (2015). Analysis of vertebral bone strength, fracture pattern, and fracture location: a validation study using a computed tomography-based nonlinear finite element analysis. Aging Dis. 6, 180–187. 10.14336/AD.2014.0621 26029476 PMC4441400

[B20] Kettler A. Schmoelz W. Shezifi Y. Ohana N. Ben-Arye A. Claes L.. (2006). Biomechanical performance of the new BeadEx implant in the treatment of osteoporotic vertebral body compression fractures: restoration and maintenance of height and stability. Clin. Biomech. 21, 676–682. 10.1016/j.clinbiomech.2006.02.005 16567025

[B21] Klazen C. A. Lohle P. N. de Vries J. Jansen F. H. Tielbeek A. V. Blonk M. C.. (2010). Vertebroplasty versus conservative treatment in acute osteoporotic vertebral compression fractures (Vertos II): an open-label randomised trial. Lancet 376, 1085–1092. 10.1016/S0140-6736(10)60954-3 20701962

[B22] La Barbera L. Cianfoni A. Ferrari A. Distefano D. Bonaldi G. Villa T. (2019). Stent-screw assisted internal fixation of severe lytic spinal metastases: a comparative finite element analysis on SAIF technique. World Neurosurg. 128, e370–e377. 10.1016/j.wneu.2019.04.154 31029814

[B23] La Barbera L. Costa F. Villa T. (2016a). ISO 12189 standard for the preclinical evaluation of posterior spinal stabilization devices – II: a parametric comparative study. Proc. Inst. Mech. Eng. H 230, 134–144. 10.1177/0954411915621588 26673809

[B24] La Barbera L. Galbusera F. Wilke H. J. Villa T. (2016b). Preclinical evaluation of posterior spine stabilization devices: can the current standards represent basic everyday life activities? Eur. Spine J. 25, 2909–2918. 10.1007/s00586-016-4622-1 27236658

[B25] La Barbera L. Galbusera F. Wilke H. J. Villa T. (2017). Preclinical evaluation of posterior spine stabilization devices: can we compare *in vitro* and *in vivo* loads on the instrumentation? Eur. Spine J. 26, 200–209. 10.1007/s00586-016-4766-z 27637903

[B26] La Barbera L. Ottardi C. Villa T. (2015). Comparative analysis of international standards for the fatigue testing of posterior spinal fixation systems: the importance of preload in ISO 12189. Spine J. 15, 2290–2296. 10.1016/j.spinee.2015.07.461 26235467

[B27] La Barbera L. Villa T. (2016). ISO 12189 standard for the preclinical evaluation of posterior spinal stabilization devices – I: Assembly procedure and validation. Proc. Inst. Mech. Eng. H 230, 122–133. 10.1177/0954411915621587 26679431

[B28] La Barbera L. Villa T. (2017). Toward the definition of a new worst-case paradigm for the preclinical evaluation of posterior spine stabilization devices. Proc. Inst. Mech. Eng. H 231, 176–185. 10.1177/0954411916684365 28095745

[B29] Li Y. X. Guo D. Q. Zhang S. C. Liang D. Yuan K. Mo G. Y.. (2018). Risk factor analysis for re-collapse of cemented vertebrae after percutaneous vertebroplasty (PVP) or percutaneous kyphoplasty (PKP). Int. Orthop. 42, 2131–2139. 10.1007/s00264-018-3838-6 29464371

[B30] Lin W. C. Lee Y. C. Lee C. H. Kuo Y. L. Cheng Y. F. Lui C. C.. (2008). Refractures in cemented vertebrae after percutaneous vertebroplasty: a retrospective analysis. Eur. Spine J. 17, 592–599. 10.1007/s00586-007-0564-y 18204942 PMC2295276

[B31] Lippuner K. (2003). Medical treatment of vertebral osteoporosis. Eur. Spine J. 12, S132–S141. 10.1007/s00586-003-0608-x 13680313 PMC3591820

[B32] McCormack T. Karaikovic E. Gaines R. W. (1994). The load sharing classification of spine fractures. Spine 19, 1741–1744. 10.1097/00007632-199408000-00014 7973969

[B33] Nagaraja S. Awada H. K. Dreher M. Bouck J. T. Gupta S. (2015). Effects of vertebroplasty on endplate subsidence in elderly female spines. J. Neurosurg. Spine 22, 273–282. 10.3171/2014.10.SPINE14195 25525963

[B34] Ottardi C. Galbusera F. Luca A. Prosdocimo L. Sasso M. Brayda-Bruno M.. (2016b). Finite element analysis of the lumbar destabilization following pedicle subtraction osteotomy. Med. Eng. Phys. 38, 506–509. 10.1016/j.medengphy.2016.02.002 26968784

[B35] Ottardi C. La Barbera L. Pietrogrande L. Villa T. (2016a). Vertebroplasty and kyphoplasty for the treatment of thoracic fractures in osteoporotic patients: a finite element comparative analysis. J. Appl. Biomater. Funct. Mater. 14, e197–204. 10.5301/jabfm.5000287 27032865

[B36] Palanca M. Barbanti-Bròdano G. Cristofolini L. (2018). The size of simulated lytic metastases affects the strain distribution on the anterior surface of the vertebra. J. Biomech. Eng. 10.1115/1.4040587 [Epub ahead of print]. 30029268

[B37] Rohlmann A. Boustani H. N. Bergmann G. Zander T. (2010). A probabilistic finite element analysis of the stresses in the augmented vertebral body after vertebroplasty. Eur. Spine J. 19, 1585–1595. 10.1007/s00586-010-1386-x 20361339 PMC2989288

[B38] Rohlmann A. Zander T. Rao M. Bergmann G. (2009). Realistic loading conditions for upper body bending. J. Biomech. 42, 884–890. 10.1016/j.jbiomech.2009.01.017 19268291

[B39] Rotter R. Heiner M. Fuerderer S. Gabl M. Roeder C. Heini P.. (2010). Vertebral body stenting: a new method for vertebral augmentation versus kyphoplasty. Eur. Spine J. 19, 916–923. 10.1007/s00586-010-1341-x 20191393 PMC2899980

[B40] Wáng Y. X. J. Deng M. He L. C. Che-Nordin N. Santiago F. R. (2018). Osteoporotic vertebral endplate and cortex fractures: a pictorial review. J. Orthop. Translat. 15, 35–49. 10.1016/j.jot.2018.08.004 30306044 PMC6169255

[B41] Wardlaw D. Cummings S. R. Van Meirhaeghe J. Bastian L. Tillman J. B. Ranstam J.. (2009). Efficacy and safety of balloon kyphoplasty compared with non-surgical care for vertebral compression fracture (FREE): a randomised controlled trial. Lancet 373, 1016–1024. 10.1016/S0140-6736(09)60010-6 19246088

[B42] White A. Panjabi M. (1990). Physical Properties and Functional Biomechanics of the Spine, in Clinical Biomechanics of the Spine. Philadelphia, PA: Lippincott Williams and Wilkins.

[B43] Wolfram U. Schwiedrzik J. (2016). Post-yield and failure properties of cortical bone. Bonekey Rep. 5:829. 10.1038/bonekey.2016.60 27579166 PMC4996317

